# The association between intolerance of uncertainty and academic burnout among university students: the role of self-regulatory fatigue and self-compassion

**DOI:** 10.3389/fpubh.2024.1441465

**Published:** 2024-07-24

**Authors:** Jingyu Qiang, Xiaowen He, Zheng Xia, Jing Huang, Cheng Xu

**Affiliations:** ^1^School of Foreign Languages, Guangdong University of Petrochemical Technology, Maoming, Guangdong, China; ^2^School of Psychology and Cognitive Science, East China Normal University, Shanghai, China; ^3^School of Business, East China University of Science and Technology, Shanghai, China

**Keywords:** intolerance of uncertainty, academic burnout, self-regulatory fatigue, self-compassion, university students

## Abstract

**Introduction:**

Increased uncertainty is a major feature of the current society that poses significant challenges to university students' mental health and academics. However, current research has not paid sufficient attention to this issue, and no study has explored the underlying mechanisms between intolerance of uncertainty and academic burnout among university students.

**Methods:**

This study examined the association between uncertainty intolerance and academic burnout among university students and the role of self-regulatory fatigue and self-compassion in light of the theory of limited resources. Convenience sampling was used to survey 1,022 Chinese university students.

**Results:**

The findings demonstrated that intolerance of uncertainty significantly influenced university students' academic burnout with self-regulatory fatigue serving as a key mediator. Additionally, self-compassion can effectively moderate the effects of intolerance of uncertainty on self-regulatory fatigue and academic burnout.

**Discussion:**

These results indicated that the depletion of cognitive resources brought about by uncertainty in the current highly uncertain social environment may be one of the key pathways to academic burnout among university students. Furthermore, current research provides insights into how to mitigate the negative effects of uncertainty on university students.

## Introduction

Since the outbreak of the COVID-19 in 2020, its highly contagious nature, severe sequelae, and disruptions to production and education caused by sudden social lockdowns and quarantines have exacerbated uncertainty in all aspects around the world (1–3). Although the current global pandemic has stabilized, the increase in overall social uncertainty caused by it has had a prolonged effect on individual mental well-being (4), bringing about anxiety, depression, stress, post-traumatic stress, and other negative consequences (5, 6). University students are among the most affected groups in the current context of social uncertainty. The university student group is in an important life stage, developing into independent adults. They are faced with multiple developmental tasks, such as interpersonal adaptation, identity, and career planning, which pose challenges to their mental health and personal development (7–9). A large body of literature suggests that the brain' s prefrontal cortex—the area involved in self-control, delaying gratification, and resisting temptation—is not fully developed until around age 25 (10). This makes them more susceptible to the temptation of instant gratification and less likely to stick to long-term goals when faced with uncertainty in life. Meanwhile, they face multiple developmental tasks, such as interpersonal adaptation, identity, and career planning, which pose challenges to their mental health and personal development (7). Moreover, the uncertainty in the social environment and labor market makes it difficult for students to set clear academic and career goals, leading to many developmental problems in university students' learning motivation (11). Unexpected problems such as the lockdown of campus life, changes in teaching models, and employment difficulties during the pandemic have intensified students' feelings of uncertainty about the environment (12–14), bringing about stress and uneasiness affecting the physical and mental health of adolescents (15, 16). However, the uncertainty in the social environment and job market makes it difficult for students to set clear academic and career goals, leading to many developmental problems in university students' learning motivation (11). Additionally, research highlights that, compared to other groups, university students are often more prone to psychological and behavioral problems due to various pressures such as academics, employment, and interpersonal relationships (17). In their book *The Coddling of the American Mind*, Lukianoff and Haidt define safetyism as a culture or belief system in which safety has become a sacred value and argue that embracing the culture of safetyism has interfered with young people's social, emotional, and intellectual development. In such an overprotective environment, young people may be afraid to face conflict, challenge or uncertainty (18). Therefore, it is of great significance to pay further attention to the negative impact of uncertainty on university students and its underlying mechanisms at present.

However, in reviewing existing research, we found that only a few researches have studied the relationship between intolerance of uncertainty and academic burnout, and there is a lack of discussion of the underlying influencing mechanisms. In the resource limitation model, self-control resources are regarded as a limited cognitive resources, and the intolerance of uncertainty will make individuals feel more stress and negative emotions in the current social environment full of uncertainty, which will continuously deplete their self-control resources (19) and ultimately have a negative impact on their psychological state and behavior. Based on the perspective of limited resources, this study explored the negative consequences of Intolerance of uncertainty on academic burnout among university students. Besides, it examined the protective factors on this basis, with a view to providing some insights to promote students' academic development and enhance their mental health.

Intolerance of uncertainty is defined as a cognitive bias that affects how a person perceives, interprets, and reacts to uncertain situations (20). Intense anxiety, worry and nervousness are common manifestations of individuals who cannot tolerate uncertainty when faced with uncertain situations. Such emotions may lead individuals to be overly concerned about the uncertainty and potential risks, which, in turn, affects their behavior and thinking patterns. Higher levels of intolerance of uncertainty have been found to affect individuals through three dimensions—cognitive, emotional, and behavioral—and are reflected in various aspects of individual functioning. For example, people with high intolerance of uncertainty levels perceive possible negative future events as stressful and view uncertainty as negative, both of which can have serious consequences and affect their performance in uncertain situations (21). Besides, many studies have confirmed that high levels of uncertainty are important factors that trigger negative emotions (22). A another recent study also points to increased uncertainty in the wider social environment as a key cause of risk perception, anxiety and rumination behavior among university students (5). Additionally, since individuals with high intolerance of uncertainty may be more likely to focus on potential risks and negative consequences, they are more inclined to adopt strategies to avoid uncertainty and risk. These behavioral patterns may further exacerbate the anxiety and worry of individuals, trapping them in a vicious cycle (23). Currently, all aspects of society are characterized by a high level of uncertainty, which has a negative effect on various populations, especially the university student population.

Academic burnout is often defined as a three-factor psychological syndrome characterized by a state of exhaustion due to the demands of coursework, a cynical and supercilious attitude toward a university degree, and a sense of reduced efficacy and academic achievement (24). Specifically, symptoms of academic burnout include high levels of anxiety, a lack of enthusiasm for learning, often accompanied by feelings of frustration and tension, apathy, lack of engagement and interest in academics, negative evaluations of oneself, a diminished sense of self-meaning and worth, and an inability to experience a sense of fulfillment from one's studies (24). One study divide the influencing factors of academic burnout into external factors and internal factors. External factors include school factors (learning pressure, teaching environment, interpersonal relationship), family factors (parenting style, parental support, family economic status). Internal factors include personality, self-esteem, and attribution style (25). Also, stress from uncertainty of future development, interpersonal relationship, and self-identity could jointly predict student academic burnout (26). Uncertainty has been found to significantly impacts the holistic development of university students. The intolerance for uncertainty situations in the face of complex social environments, academic pressures, and future uncertainty leads to a sense of loss of control and powerlessness among university students (5), which is likely to affect their academic performance and psychological state. Several studies indicated that uncertainty stress not only leads to poorer self-rated health, but is also closely related to poorer academic performance (27, 28). Additionally, low tolerance for uncertainty is closely related to feelings of low efficacy (29). Both of these are likely to lead to feelings of fatigue and helplessness, exacerbating the state of academic burnout (30). Thus, intolerance of uncertainty may be significantly positively influenced academic burnout.

Self-regulatory fatigue is a persistent state caused by chronic resource depletion associated with the depletion of self-control resources (31). Research indicates that behaviors related to self-control deplete cognitive resources and trigger ego depletion (32). Individuals with high intolerance of uncertainty are more sensitive to uncertain events in their lives and tend to be more negative as well, and are therefore more likely to exhibit stronger stress responses (33). A study of medical students also noted a strong correlation between intolerance of uncertainty and symptoms of worry about the future and burnout (34). These suggest that in the face of stressful and negative situations, individuals consume a significant amount of cognitive resources to cope with events in order to minimize uncertainty and regulate the ensuing negativity, which may trigger self-regulatory fatigue. When individuals are in a state of high cognitive resource depletion, which negatively affects their coping ability, self-control, and other aspects of coping, they tend to prefer negative avoidance coping strategies and experience more failures in self-control (31, 35). This implies that students who are motivated by a state of self-regulation fatigue may have more difficulty coping with academic difficulties and sustaining engagement in their studies, and be prone to have negative attitudes, feel frustrated and anxious, and lose interest in their studies. Therefore, self-regulatory fatigue may mediate the effects of intolerance of uncertainty on academic burnout.

Self-compassion refers to an individual's understanding and relief in a positive and kind manner, firmly believing that they deserve love and happiness even in the face of failure and pain (36). Individuals with higher levels of self-compassion tend not to criticize themselves or avoid pain, even when they suffer setbacks and misfortune, but rather maintain a forgiving attitude, seeing them as shared, universal human experiences and unconditionally accepting all that they are (37). In the field of mental health, self-compassion is recognized as an important protective factor and can be effective in improving and enhancing an individual's mental health (38). Self-compassion can also help alleviate the negative emotions through adaptive strategies, such as increasing self-kindness and positive thoughts and reducing self-criticism (39). Additionally, adolescents with high self-compassion have higher resilience in the face of adversity, a greater sense of efficacy, and a greater sense of academic control (40). A longitudinal survey also pointed out that the level of self-compassion has a significant positive impact on the academic performance of university students (41). Therefore, self-compassion may be able to directly mitigate the negative effects of intolerance of uncertainty on academic burnout.

Moreover, studies have indicated that self-compassion is an important psychological resource for individuals to cope with uncertainty and threats (42), and levels of self-compassion are strongly correlated with positive emotions (37). It has been found that inducing positive emotions can help individuals restore their willingness and ability to self-regulate. Additionally, people with high levels of self-compassion tend not to resort to negative emotion regulation and do not tend to repress their emotional responses (37). They experience negative events more cautiously and less reactively, and because they are less negatively affected, they may not need to expand their significant self-regulatory resources to manage their emotions. Thus, self-compassion may moderate the effects of intolerance of uncertainty on self-regulatory fatigue.

**Figure 1:**
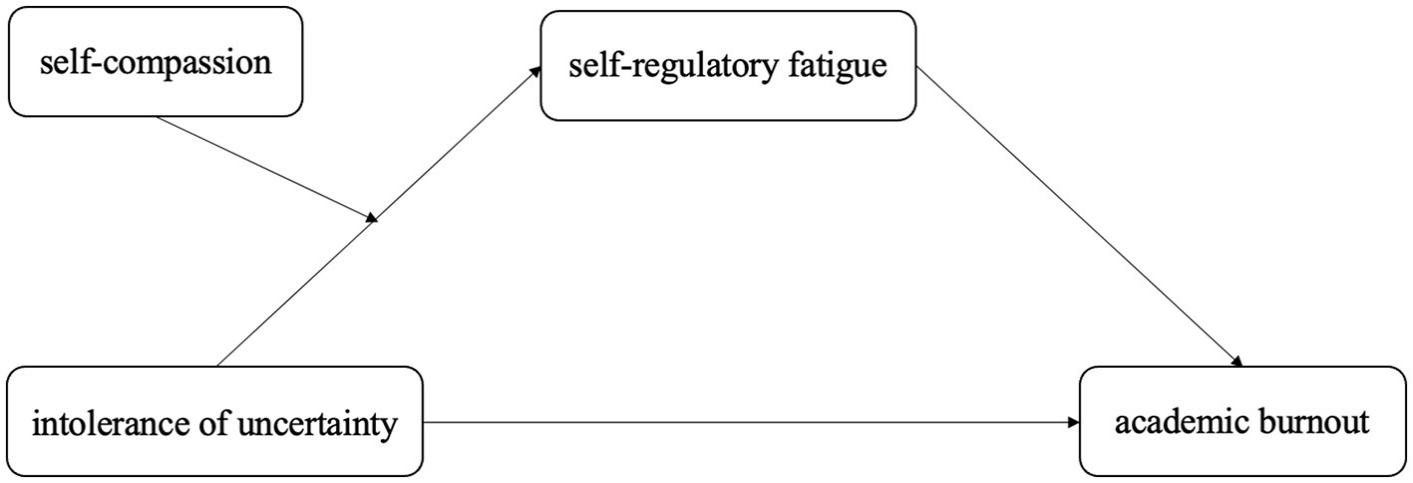
Proposed model.

To summarize, this study established a moderated mediated-effects model (Figure 1) to test the effects of uncertainty on university students' academic burnout and its intermediate mechanisms in the current social environment and proposed the following hypotheses:

1. Intolerance of uncertainty can significantly predict the level of university students' academic burnout.
2. Self-regulatory fatigue mediates between intolerance of uncertainty and academic burnout.
3. Self-compassion moderates the effect of intolerance of uncertainty on academic burnout.
4. Self-compassion moderated the effect of intolerance of uncertainty on self-regulatory fatigue.

## Methods

### Participants

In this study, 1,154 questionnaires were distributed using convenience sampling, and 132 invalid (not answering carefully, omitting answers, and regularity of answers) questionnaires were excluded. Finally, 1,022 valid questionnaires were obtained with a validity rate of 88.56%. Among them, 508 (49.70%) were men and 514 (50.30%) were women. The ages of the participants ranged from 18–30 years (*M* = 20.19; *SD* = 2.68). There were 48 (4.70%) specialist students, 887 (86.80%) undergraduate students and 87 (8.50%) postgraduate students out of the total participants. This study was approved by the ethics committee of the organization where it was conducted (No: 20230912001). We distributed the questionnaire through an online questionnaire platform. The informed consent text is included at the beginning of the questionnaire. All the respondents granted permission for inclusion by reading the introductions for the investigation and provided voluntary response.

### Instrument

#### Intolerance of Uncertainty Scale

The Intolerance of Uncertainty Scale was used to measure the degree to which individuals tolerated uncertain events (43). The scale has 12 items, such as “Unpredictable events make me upset” and “Uncertainty prevents me from having a fulfilling life”, on a 5-point Likert scale ranging from 1 (not at all) to 5 (completely). The higher the total score, the greater the intolerance of uncertainty the individual has. Cronbach's alpha coefficient was 0.92 for the scale used in this study.

#### Self-Regulatory Fatigue Scale

The Self-Regulatory Fatigue Scale developed by Nes et al. and modified by Wang et al. (31, 44) was used to assess self-regulatory fatigue. The scale has 18 items, including “I feel energetic” and “I have difficulty executing my exercise programme”. The scale consists of three subscales: cognitive, emotional, and behavioral. A 5-point Likert scale ranging from 1 (strongly disagree) to 5 (strongly agree) was used. The higher the total score, the greater the degree of self-regulatory fatigue. Questions 1, 2, 5, 9, and 14 were reverse-scored. In this study, the Cronbach's alpha was 0.88. The alpha coefficients for the cognitive, emotional, and behavioral subscales were 0.80, 0.77, and 0.80, respectively.

#### Academic Burnout Scale

The Academic Burnout Scale developed by Lian et al. (45) was used to assess academic burnout among university students. The scale consists of 20 items, such as “I feel that what I have learned is useless” and “I am tired of studying”. The scale has three dimensions: dejection, improper behavior, and reduced personal accomplishment. All items are scored on a 5-point Likert scale ranging from 1 (not at all) to 5 (completely). The higher the total score, the higher the level of academic burnout. Questions 1, 3, 6, 8, 11, 13, 15, and 18 were reverse-scored. Cronbach's alpha coefficient for the scale was 0.88, and the alpha coefficients for the three subscales of dejection, improper behavior, and reduced personal accomplishment were 0.89, 0.74, and 0.79, respectively.

#### Self-Compassion Scale

The Self-Compassion Scale developed by Neff (46) and revised by Chen et al. (47) was used to assess the level of self-compassion among university students. The scale consists of 26 items, such as “I am dissatisfied with and critical of my own shortcomings and deficiencies” and “I try to face painful things with a calm mind when they happen”. The scale has six dimensions: self-Kindness, Self-Judgement, Common Humanity, Isolation, Mindfulness, and Over-identification. All items are scored on a 5-point Likert scale, ranging from 1 (Never) to 5 (Always). Higher total scores indicate higher levels of self-compassion. Cronbach's alpha coefficient for the scale in this study was 0.82, and the alpha coefficients of the six dimensions of Self-Kindness, Self-Judgement, Common Humanity, Isolation, Mindfulness, and Over-identification were 0.87, 0.81, 0.77, 0.79, 0.82, and 0.79, respectively.

## Results

### Common method bias

As the data were collected using self-reported methods, the results may be affected by common method bias. The Harman one-way test (48) was used to test for common method bias. A total of 13 common factors with eigenvalues >1 were obtained without rotation, of which the first one had an explanatory rate of 21.26%, which was much lower than 40%, indicating that there is no serious common method bias.

### Results of descriptive statistics and correlation analysis among variables

Table 1 shows the results of the descriptive statistics and correlation analyses of the study variables. Intolerance of uncertainty was positively correlated significantly with self-regulatory fatigue and academic burnout and negatively correlated significantly with self-compassion. Self-regulatory fatigue and self-compassion were significantly negatively correlated and significantly positively correlated with academic burnout. Academic burnout and self-compassion were significantly and negatively correlated.

**Table 1:** Descriptive statistics and correlation analysis of variables.

**Variable**	***M***	***SD***	**1**	**2**	**3**	**4**
1. Intolerance of uncertainty	34.25	10.01	—			
2. Self- regulatory fatigue	47.90	10.30	0.54^**^	—		
3. Academic burnout	60.43	10.55	0.39^**^	0.52^**^	—	
4. Self-compassion	80.47	10.52	−0.16^**^	−0.37^**^	−0.31^**^	—

N = 1,022. ^**^p < 0.01.

The results of the independent samples *t*-test showed significant differences in intolerance of uncertainty (*t* = 12.24, *p* < 0.001), self-regulatory fatigue (*t* = 6.20, *p* < 0.001), and academic burnout (*t* = 4.49, *p* < 0.001) among university students of different genders. One-way ANOVA results showed significant differences in intolerance of uncertainty, F_(12, 1, 009)_ = 9.49, *p* < 0.001, self-regulatory fatigue, *F*_(12, 1, 009)_ = 8.65, *p* < 0.001, and academic burnout, *F*_(12, 1, 009)_ = 3.03, *p* < 0.001 among university students of different age groups. Home location (urban, rural, or township) had a significant effect on intolerance of uncertainty, *F*_(12, 1, 009)_ = 17.37, *p* < 0.001, and self-regulatory fatigue, *F*_(12, 1, 009)_ = 6.81, *p* < 0.001. Additionally, family economic status had a significant effect on intolerance of uncertainty, *F*_(12, 1, 009)_ = 5.66, *p* < 0.001 and self-regulatory fatigue, *F*_(10, 1011)_ = 2.10, *p* = 0.022. Therefore, we included gender, age, home location, and household economic status as control variables in the model.

### Mediation model

Model 4 from the Process Macro programme developed by Hayes (49) was used to test the mediation model. Gender, age, home location, and family economic level were included as control variables in the model, considering their effects on the study variables. As shown in Table 2, intolerance of uncertainty positively predicted academic burnout (β = 0.39, *p* < 0.001). When self-regulatory fatigue was introduced as a mediating variable, intolerance of uncertainty remained a significant predictor of academic burnout (β = 0.16, *p* < 0.001), and intolerance of uncertainty also significantly predicted self-regulatory fatigue (β = 0.52, *p* < 0.001). Self-regulatory fatigue also predicted academic burnout at a significant level (β = 0.46, *p* < 0.001). The results of the mediation test for self-regulatory fatigue (see Table 3) showed that the bootstrap 95% confidence interval did not contain a zero between the upper and lower bounds, indicating that self-regulatory fatigue mediated the effect of intolerance of uncertainty on academic burnout, with a mediation effect of 0.24, which accounted for 61.54% of the total effect.

**Table 2:** Mediation model test for self-regulatory fatigue.

**Regression equation**		**Overall fit indices**			**Significance of the regression coefficients**		
**Outcome variables**	**Predictors**	***R***	***R** ^2^*	***F***	β	***95%CI***	***t***
Academic burnout	Intolerance of uncertainty	0.39	0.15	36.13^***^	0.39	[0.33, 0.45]	12.29^***^
Self-regulatory fatigue	Intolerance of uncertainty	0.57	0.31	91.13^***^	0.52	[0.46, 0.57]	18.05^***^
Academic burnout	Intolerance of uncertainty	0.54	0.29	70.43^***^	0.16	[0.09, 0.22]	4.66^***^
	Self-regulatory fatigue				0.46	[0.39, 0.52]	14.37^***^

N = 1022, ^***^p < 0.001. Gender, age, home location and economic level were done as control variables included in the model. The study variables were standardized.

**Table 3:** Mediation model effect size analysis for self-regulatory fatigue.

	**Effect**	**SE**	**95%CI**
Total effect	0.39	0.03	[0.33, 0.45]
Direct effect	0.16	0.03	[0.09, 0.22]
Indirect effect of self-regulatory fatigue	0.24	0.03	[0.19, 0.29]

B, Effect size. SE and 95% CI refer to the standard errors, lower and upper 95% confidence intervals of the indirect effects estimated by the bias-corrected percentile Bootstrap method, respectively.

### Moderated mediation model

Model 8 in the Process Macro programme developed by Hayes, was used to test the moderated mediation model. Intolerance of uncertainty and self-compassion significantly predicted academic burnout after controlling for gender, age, home location, and family economic status. Additionally, the interaction between intolerance of uncertainty and self-compassion significantly predicted individuals' levels of self-regulatory fatigue (Table 4).

**Table 4:** Moderated mediation model test.

**Regression equation**		**Overall fit indices**			**Significance of the regression coefficients**		
**Outcome variables**	**Predictors**	***R***	***R** ^2^*	***F***	β	***95%CI***	***t***
Self-regulatory fatigue	Intolerance of uncertainty	0.64	0.40	98.30^***^	0.48	[0.42, 0.53]	17.54^***^
	Self-compassion				−0.25	[−0.30, −0.20]	−9.33^***^
	Intolerance of uncertainty ^*^ self-compassion				−0.10	[−0.15, −0.05]	−4.14^***^
Academic burnout	Intolerance of uncertainty	0.57	0.32	60.22^***^	0.18	[0.12, 0.25]	5.49^***^
	Self-regulatory fatigue				0.38	[0.31, 0.45]	11.32^***^
	Self-compassion				−0.09	[−0.15, −0.04]	−3.16^**^
	Intolerance of uncertainty ^*^ self-compassion				−0.11	[−0.16, −0.06]	−4.24^***^

N = 1022, ^**^p < 0.01, ^***^p < 0.001. Gender, age, home location and economic level were done as control variables included in the model. The study variables were standardized.

**Figure 2:**
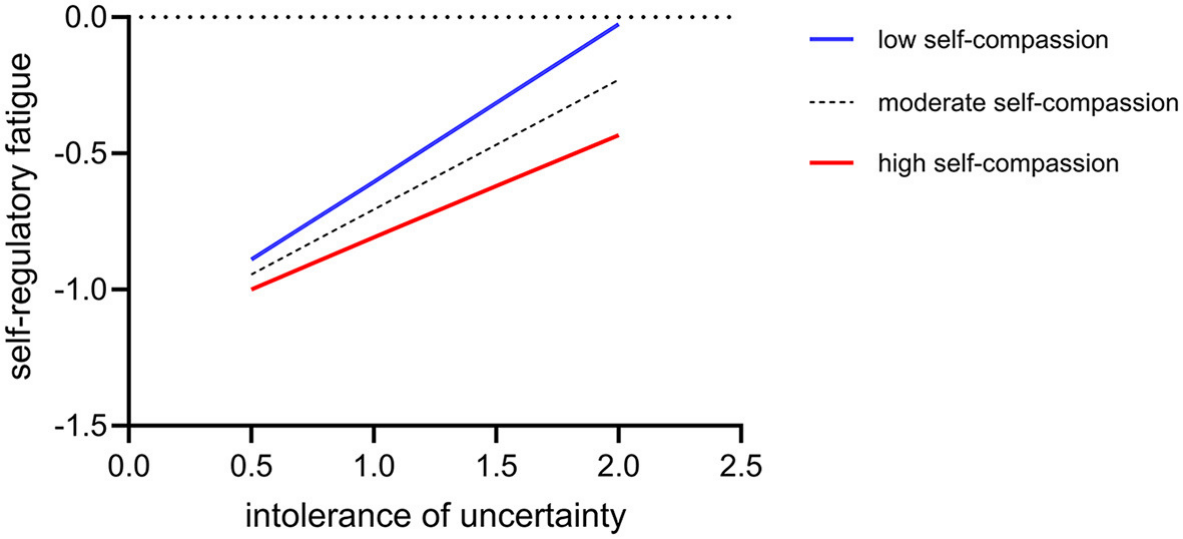
The moderating role of self-compassion between intolerance of uncertainty and self-regulatory fatigue.

To illustrate the moderating effect of self-compassion better, we conducted a simple slope analysis. Figure 2 shows that for participants with lower self-compassion (M-SD), intolerance of uncertainty significantly and positively predicted self-regulatory fatigue (simple slope = 0.29, *p* < 0.001). In contrast, for participants with high self-compassion (M + SD), the predictive effect of intolerance of uncertainty on self-regulatory fatigue was not significant (simple slope = 0.07, *p* = 0.092).

**Figure 3:**
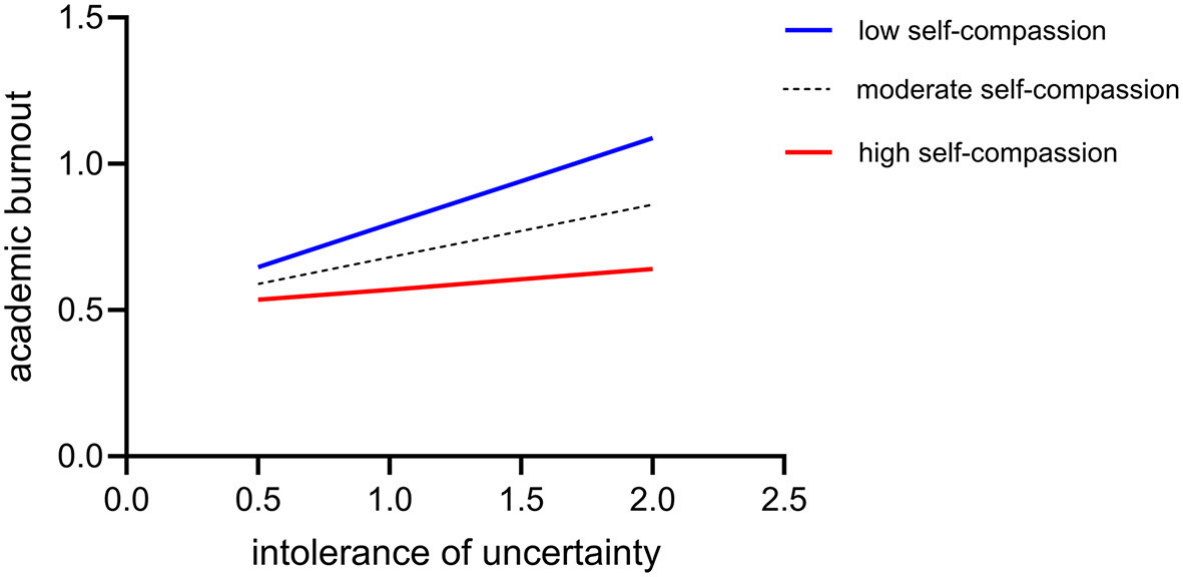
The moderating role of self-compassion between intolerance of uncertainty and academic burnout.

Figure 3 shows that for participants with low self-compassion (M-SD), intolerance of uncertainty significantly and positively predicted academic burnout (simple slope = 0.58, *p* < 0.001), whereas for participants with high self-compassion (M + SD), intolerance of uncertainty also significantly and positively predicted academic burnout. However, its predictive effect was smaller (simple slope = 0.38, *p* < 0.001). This finding suggests that the effect of uncertainty intolerance on academic burnout decreases significantly as self-compassion increases.

## Discussion

Based on the current context of social uncertainty, this study explored the impact of intolerance of uncertainty on university students' academic burnout and explored the mechanisms and protective factors. We found that intolerance of uncertainty not only directly affects university students' academic burnout but also further exacerbates the level of academic burnout by causing individuals to experience self-regulatory fatigue. Additionally, self-compassion is an effective protective factor that can mitigate the negative effects of uncertainty.

### Intolerance of uncertainty and academic burnout

This study found that individuals' intolerance of uncertainty positively predicted academic burnout, supporting Hypothesis 1. This result may be related to the stress response, negative emotions, and lower self-efficacy experienced by individuals facing uncertainty. Uncertainty in life situations can have a negative impact on individuals' emotions (50). Individuals who are unable to tolerate uncertainty are more sensitive to uncertain environments in which they experience stronger psychological and behavioral responses, such as anxiety and a sense of being out of control (5, 29), hindering students' positive engagement in their studies. Besides, students with higher levels of intolerance of uncertainty may feel more stress in the face of academic challenges, possibly from worrying about unknown outcomes or the psychological discomfort of being in an unknown situation. When this stress accumulates, and individuals lack sufficient psychological resources to cope with it, it can trigger strong feelings of powerlessness and lower self-efficacy, which in turn affects their engagement in academics and determination (51), and students with lower self-efficacy may tend to avoid academic challenges and develop negative learning behaviors such as procrastination. Additionally, uncertainty means that individuals lack the information necessary to make decisions (52) and may activate an individual's behavioral inhibitory system, which inhibits relevant actions in the face of complexity and uncertainty to reduce the risk of harm (53). Thus, university students who cannot tolerate uncertainty are more likely to inhibit their actions and reduce their academic engagement when faced with uncertainty.

### The mediating role of self-regulatory fatigue

Intolerance of uncertainty not only directly affects academic burnout but also impacts academic burnout through self-regulatory fatigue, supporting Hypothesis 2, suggesting that the great depletion of cognitive resources may be an important mechanism for individuals to develop academic burnout in uncertainty. University students themselves face stressful situations from many perspectives, such as future career opportunities, interpersonal relationships, and personal achievements, as well as anxiety and stress triggered by the uncertainty of these scenarios. Students who cannot tolerate higher levels of uncertainty may need to consume more psychological resources for self-control and emotion regulation when coping with uncertain environments, leading to self-regulatory fatigue. Additionally, previous research has proposed that since both self-control and cognition-related tasks require individuals to activate and regulate controlled brain regions, performing self-control or cognitive processing consumes a large amount of energy and produces psychological fatigue, ultimately reducing the level of motivation to perform the task (54). Additionally, high levels of intolerance of uncertainty may have triggered excessive stress responses and maladaptive self-regulation in university students beyond their own abilities and resources, manifesting as mood swings, inattention, and decreased decision-making ability, which affects an individual's overall mental health and resilience (5, 55), which in turn has a negative effect on their behavior and mental state, leading to higher levels of academic burnout (56, 57). Low tolerance for uncertainty, as opposed to high tolerance for uncertainty, requires students to continually call upon more psychological resources to adjust their state and cope with important events in their lives, thereby increasing the likelihood of self-regulatory fatigue.

### The moderating role of self-compassion

The results showed that self-compassion played a moderating role in the relationship between intolerance of uncertainty and academic burnout, supporting Hypothesis 3, further supporting the emotion regulation model of self-compassion, which is effective in alleviating individuals' negative feelings in unfavorable situations as well as the ensuing problem of academic burnout. Moreover, self-compassion is highly correlated with an individual's adaptive coping strategies, and university students with high levels of self-compassion are likely to exhibit more adaptive behavioral styles of proactive coping (58), actively seeking help and reducing uncertainty. Moreover, when faced with unfavorable situations brought about by uncertainty, they are more inclined to self-acceptance and can recognize that this is a status quo that the whole society is facing, not just their own encounters. Thus, they are able to remain calm and avoid the emergence of undesirable grounded behavioral patterns and are less likely to show negative attitudes (59). In contrast, university students with low self-compassion showed poorer coping styles, such as self-blame, rumination, and avoidance, and, therefore, more academic burnout.

This study also found that self-compassion plays a moderating role in the relationship between intolerance of uncertainty and self-regulation fatigue, supporting Hypothesis 4. Individuals with high levels of self-compassion are able to view their experiences with a friendly, open, and tolerant attitude, even when faced with great uncertainty. They are better able to understand the situation at hand, thus avoiding the constant consumption of intrinsic resources on negative experiences and negative emotions. Additionally, individuals with high levels of self-compassion tend to experience more positive emotions (38). According to the Extension and Construction Theory of Positive Emotions, positive emotions can help individuals build up more permanent personal resources that serve as a psychological reserve to help them cope with stressful and negative situations (60). Thus suggesting that self-compassion can provide individuals with more intrinsic supportive energy, effectively avoiding self-regulatory fatigue due to uncertainty.

### Implications

The current society is facing unprecedented uncertainty, and the New Crown epidemic (COVID-19), as a major event in recent years, has also brought about far-reaching negative psychological impacts on people. Research has shown that this psychological impact may last for more than 10 or 20 years (61). University students, an important group in society, are in an important transition and critical period in their lives, and the uncertainty of the environment may have great negative consequences for them. Based on the current context of uncertainty in society, this study investigated the negative effects of intolerance of uncertainty on university students' academic performance and pointed out that the depletion of cognitive resources might be the key pathway of uncertainty leading to academic burnout. Additionally, the importance of self-compassion in mitigating the effects of uncertainty was confirmed. It also provides insights into improving the mental health and academic performance of university students. For example, in the face of uncertain environments and pressures, a more accepting and accommodating attitude is adopted to avoid the constant consumption of cognitive resources during negative emotions. In addition, universities and society should pay more attention to the impact of the current uncertain environment on students. They can encourage more gaming in schools during adolescence, limit teenagers' screen time, provide more free play and rest time, carry out more vocational education and youth development programs, and for college students facing employment pressure and fierce competition, while providing necessary psychological support and counseling, they should also increase college students' participation in the real world and their integration into the community, such as providing more practical internships and employment training.

### Limitations and prospects

However, this study had some limitations. First, the cross-sectional research design used failed to reveal causal relationships between the variables. Future research could adopt a longitudinal tracking design to explore the factors influencing university students' academic burnout in greater depth. Second, the data were collected using self-report questionnaires, which have a subjective reporting bias and may have affected the reliability of the results. In the future, this could be comprehensively assessed by combining interviews with assessments of other people. Third, while this study focused on university students, research shows that both primary and secondary school students currently suffer from serious academic burnout and other problems (62). Does uncertainty have an impact on academic burnout in younger adolescents? Further validation of the model in other groups is required.

## Conclusions

Intolerance of uncertainty significantly predicts the level of academic burnout in university students. Self-regulatory fatigue mediated the effect of intolerance of uncertainty on academic burnout. Self-compassion moderates the effect of intolerance of uncertainty on academic burnout. Self-compassion moderated the effect of intolerance of uncertainty on self-regulatory fatigue.

## Data availability statement

The raw data supporting the conclusions of this article will be made available by the authors, without undue reservation.

## Ethics statement

The studies involving humans were approved by Ethics Committee of Guangdong University of Petrochemical Technology. The studies were conducted in accordance with the local legislation and institutional requirements. Written informed consent for participation was not required from the participants or the participants' legal guardians/next of kin because the questionnaire was distributed through an online platform, and although there was no paper-based informed consent form, the study included an online informed consent process, whereby all participants were shown a paragraph about the informed consent instructions before they were invited to fill out the questionnaire, which included all the elements that should be included in the paper-based version of the informed consent form, such as the content of the study, participant's rights and interests, and a description of the risks. Participants confirmed their willingness to participate in the study. All the participants granted permission for inclusion by reading the introductions for the investigation and provided voluntary response. The informed consent process on this line was approved by the Ethics Committee of the host institution.

## Author contributions

JQ: Conceptualization, Data curation, Formal analysis, Funding acquisition, Investigation, Methodology, Resources, Supervision, Validation, Visualization, Writing – original draft. XH: Formal analysis, Investigation, Methodology, Software, Writing – original draft, Writing – review & editing. ZX: Data curation, Methodology, Writing – original draft. JH: Conceptualization, Methodology, Writing – original draft. CX: Conceptualization, Data curation, Formal analysis, Funding acquisition, Investigation, Methodology, Project administration, Resources, Software, Supervision, Validation, Visualization, Writing – original draft, Writing – review & editing.

## References

[1] Altig D Baker S Barrero JM Bloom N Bunn P Chen S. Economic uncertainty before and during the COVID-19 pandemic. J Public Econ. (2020) 191:104274. 10.1016/j.jpubeco.2020.104274 32921841 PMC7480328

[2] Tull MT Barbano AC Scamaldo KM Richmond JR Edmonds KA Rose JP. The prospective influence of COVID-19 affective risk assessments and intolerance of uncertainty on later dimensions of health anxiety. J Anxiety Disord. (2020) 75:102290. 10.1016/j.janxdis.2020.102290 32823216 PMC7422821

[3] Xu C Lin N Shen Z Xie Z Xu D Fu J. Bedtime procrastination related to loneliness among Chinese university students during post-pandemic period: a moderated chain mediation model. BMC Public Health. (2024) 24:1–13. 10.1186/s12889-024-18019-6 38365682 PMC10870653

[4] Evers K Gijbels E Maljaars J Rumball F Spain D Happé F. Mental health of autistic adults during the COVID-19 pandemic: The impact of perceived stress, intolerance of uncertainty, coping style. Autism. (2023) 27:832–847. 10.1177/13623613221119749 36263743 PMC9582738

[5] Fu J Xu C Yan W Li L. The effect of intolerance of uncertainty on state anxiety in the regular epidemic prevention and control phase in the context of informatization: a moderated chain mediation model. Appl Res Qual Life. (2023) 18:1849–73. 10.1007/s11482-023-10165-0 37359220 PMC10031185

[6] Xu C Yan WH. The relationship between information overload and state of anxiety in the period of regular epidemic prevention and control in China: a moderated multiple mediation model. Curr Psychol. (2023) 42:21842–59. 10.1007/s12144-022-03289-3 35693836 PMC9169442

[7] Guo H. A brief discussion on the causes and countermeasures of college students' psychological confusion and mental health problems. Rev Educ Theory. (2021) 4:3615. 10.30564/ret.v4i4.3615

[8] Jahrami H Saif Z Trabelsi K Ghazzawi H Pandi-Perumal SR Seeman MV. An umbrella review and a meta-analysis of meta-analyses of disordered eating among medical students. Alpha Psychiat. (2024) 25:165–74. 10.5152/alphapsychiatry.2024.241515 38798808 PMC11117415

[9] Jia X Huang Y Yu W Ming WK Qi F Wu Y. A moderated mediation model of the relationship between family dynamics and sleep quality in college students: the role of big five personality and only-child status. Int J Environ Res Public Health. (2022) 19:3576. 10.3390/ijerph19063576 35329263 PMC8953608

[10] Johnson SB Blum RW Giedd JN. Adolescent maturity and the brain: the promise and pitfalls of neuroscience research in adolescent health policy. J Adolesc Health. (2009) 45:216–21. 10.1016/j.jadohealth.2009.05.016 19699416 PMC2892678

[11] Gibbons C. Surviving a pandemic - Understanding the role of student stress, personality and coping on course satisfaction and anxiety during lockdown. Innovat Educ Teach Int. (2022) 60:463–75. 10.1080/14703297.2022.2064326

[12] Joseph RA Kim JJ Akers SW Turner T Whorley E Lumpkin K. COVID-19 related stress, quality of life, and intrinsic religiosity among college students during the global pandemic: a cross-sectional study. Cogent Psychol. (2023) 10:2195091. 10.1080/23311908.2023.2195091

[13] Kareem J Thomas S Kumar PA Neelakantan M. The role of classroom engagement on academic grit, intolerance to uncertainty and well-being among school students during the second wave of the COVID-19 pandemic in India. Psychol Sch. (2023) 60:1594–608. 10.1002/pits.22758 35942393 PMC9349720

[14] Mok KH Xiong W Ye H. COVID-19 crisis and challenges for graduate employment in Taiwan, Mainland China and East Asia: a critical review of skills preparing students for uncertain futures. J Educ Work. (2021) 34:247–61. 10.1080/13639080.2021.1922620

[15] Xu C Shen Z Lin N Xie Z Xie L Wang Z. The Effect of COVID-19 Information overload on emotional eating in post-pandemic period in China: the mediating role of fear of COVID-19 and the moderating role of self-compassion. Appl Res Qual Life. (2023) 18:2935–54. 10.1007/s11482-023-10213-9

[16] Zhu Z Wu D Wei K Liu Y Xu Z Jiao G. Uncertainty stress and self-rated health during the early stage of the COVID-19 outbreak. Health Psychol Behav Med. (2023) 11:2173202. 10.1080/21642850.2023.2173202 36818392 PMC9936997

[17] Auerbach RP Alonso J Axinn WG Cuijpers P Ebert DD Green JG. Mental disorders among college students in the World Health Organization World Mental Health Surveys. Psychol Med. (2016) 46:2955–70. 10.1017/S0033291716001665 27484622 PMC5129654

[18] Lukianoff G Haidt J. The Coddling of the American Mind: How Good Intentions and Bad Ideas are Setting up a Generation for Failure. London: Penguin. (2019).

[19] Baumeister RF. Ego depletion and self-control failure: an energy model of the self's executive function. Self Ident. (2002) 1:129–36. 10.1080/152988602317319302

[20] Dugas MJ Schwartz A Francis K. Brief report: intolerance of uncertainty, worry, and depression. Cogn Ther Res. (2004) 28:835–42. 10.1007/s10608-004-0669-0

[21] Rettie H Daniels J. Coping and tolerance of uncertainty: predictors and mediators of mental health during the COVID-19 pandemic. Am Psychol. (2021) 76:427. 10.1037/amp0000710 32744841

[22] Rosser BA. Intolerance of uncertainty as a transdiagnostic mechanism of psychological difficulties: a systematic review of evidence pertaining to causality and temporal precedence. Cognit Ther Res. (2019) 43:438–63. 10.1007/s10608-018-9964-z

[23] Bavolar J Kacmar P Hricova M Schrötter J Kovacova-Holevova B Köverova M. Intolerance of uncertainty and reactions to the COVID-19 pandemic. J Gen Psychol. (2023) 150:143–70. 10.1080/00221309.2021.1922346 34006200

[24] Schaufeli WB Martinez IM Pinto AM Salanova M Bakker AB. Burnout engagement in university students: a cross-national study. J Cross Cult Psychol. (2002) 33:464–81. 10.1177/0022022102033005003

[25] Lin F Yang K. The External and Internal Factors of Academic Burnout. (2021). 10.2991/assehr.k.211220.30732175718

[26] Lin S-H Huang Y-C. Life stress and academic burnout. Active Learn High Educ. (2014) 15:77–90. 10.1177/1469787413514651

[27] Charbonnier E Le Vigouroux S Puechlong C Montalescot L Goncalves A Baussard L. The effect of intervention approaches of emotion regulation and learning strategies on students' learning and mental health. Inquiry. (2023) 60:469580231159962. 10.1177/00469580231159962 36998220 PMC10068999

[28] Wu D Yang T. Late bedtime, uncertainty stress among Chinese college students: impact on academic performance and self-rated health. Psychol Health Med. (2023) 28:2915–26. 10.1080/13548506.2022.2067337 35437084

[29] Huntley C Young B Tudur Smith C Jha V Fisher P. Testing times: the association of intolerance of uncertainty and metacognitive beliefs to test anxiety in college students. BMC Psychol. (2022) 10:1–7. 10.1186/s40359-021-00710-7 34986890 PMC8729154

[30] Larrazabal MA Naragon-Gainey K Conway CC. Distress tolerance and stress-induced emotion regulation behavior. J Res Pers. (2022) 99:104243. 10.1016/j.jrp.2022.104243

[31] Nes LS Ehlers SL Whipple MO Vincent A. Self-regulatory fatigue in chronic multisymptom illnesses: scale development, fatigue, and self-control. J Pain Res. (2013) 6:181–188. 10.2147/JPR.S40014 23526193 PMC3596127

[32] Baumeister RF Bratslavsky E Muraven M Tice DM. Ego depletion: is the active self a limited resource? J Pers Soc Psychol. (1998) 74:1252–65. 10.1037//0022-3514.74.5.1252 9599441

[33] Barling J Frone MR. If only my leader would just do something! Passive leadership undermines employee well-being through role stressors and psychological resource depletion. Stress and Health. (2017) 33:211–22. 10.1002/smi.2697 27470980 PMC5791740

[34] Johnson MW Gheihman G Thomas H Schiff G Olson APJ Begin AS. The impact of clinical uncertainty in the graduate medical education (GME) learning environment: a mixed-methods study. Med Teach. (2022) 44:1100–8. 10.1080/0142159X.2022.2058383 35666840

[35] Gao Y Shan Y Jiang T Cai L Wang H. Dietary adherence, self-regulatory fatigue and trait self-control among chinese patients with peritoneal dialysis: a cross-sectional study. Patient Pref Adher. (2021) 15:443–51. 10.2147/PPA.S298231 33658768 PMC7920602

[36] Neff KD. Self-compassion: An alternative conceptualization of a healthy attitude toward oneself. Self Identity. (2003) 2:85–101. 10.1080/15298860309032

[37] Neff KD Kirkpatrick KL Rude SS. Self-compassion adaptive psychological functioning. J Res Pers. (2007) 41:139–54. 10.1016/j.jrp.2006.03.004

[38] Neff K Germer C. Self-compassion psychological. In: The Oxford Handbook of Compassion Sciencec. New York, NY: Oxford University Press (2017). p. 371–90.

[39] Inwood E Ferrari M. Mechanisms of change in the relationship between self-compassion, emotion regulation, and mental health: a systematic review. Appl Psychol. (2018) 10:215–35. 10.1111/aphw.12127 29673093

[40] Manavipour D Saeedian Y. The role of self-compassion and control belief about learning in university students' self-efficacy. Journal of Contextual Behavioral Science. (2016) 5:121–6. 10.1016/j.jcbs.2016.02.00327409075

[41] Egan H O'hara M Cook A Mantzios M. Mindfulness, self-compassion, resiliency and wellbeing in higher education: a recipe to increase academic performance. J Furth Higher Educ. (2022) 46:301–311. 10.1080/0309877X.2021.1912306

[42] Lau HP Chan LW Ng SM. Self-compassion buffers the adverse mental health impacts of COVID-19-related threats: results from a cross-sectional survey at the first peak of Hong Kong's outbreak. Front Psychiat. (2020) 11:585270. 10.3389/fpsyt.2020.585270 33250793 PMC7674650

[43] Zhang Y Song J Gao Y Wu S Song L Miao D. (2017). Reliability and Validity of the Intolerance of Uncertainty Scale-Short form in University Students. Chinese Journal of Clinical Psychology *02(v.25)* 92–95.

[44] Wang L Zhang J Wang J Tao T Fan C Gao W. Validity reliability of the Chinese version of the Self-Regulatory Fatigue Scale in young adults. Chin Mental Health J. (2015) 29:290–4.

[45] Lian R Yang L Wu L. Relationship betwen profesional commitment and learning burnout of undergraduates and scales developing. Acta Psychologica Sinica. (2005) 5:632–636.

[46] Neff KD. The development and validation of a scale to measure self-compassion. Self Identity. (2003) 2:223–50. 10.1080/1529886030902726979311

[47] Chen J Yan L Zhou L. Reliability and validity of Chinese version of self-compassion scale. Chin J Clini Psychol. (2011) 19:734–6. 10.1038/cmi.2011.4 21358669 PMC4012873

[48] Podsakoff PM MacKenzie SB Lee J-Y Podsakoff NP. Common method biases in behavioral research: a critical review of the literature and recommended remedies. J Appl Psychol. (2003) 88:879. 10.1037/0021-9010.88.5.879 14516251

[49] Hayes AF. Introduction to Mediation, Moderation, and Conditional Process Analysis: A Regression-Based Approach. New York: Guilford Publications. (2017).

[50] Morriss J Goh K Hirsch CR Dodd HF. Intolerance of uncertainty heightens negative emotional states and dampens positive emotional states. Front Psychiatry. (2023) 14:1147970. 10.3389/fpsyt.2023.1147970 37032949 PMC10073686

[51] Uzun K Karataş Z. Predictors of academic self efficacy: intolerance of uncertainty, positive beliefs about worry and academic locus of control. Int Educ Stud. (2020) 13:104. 10.5539/ies.v13n6p104

[52] Bar-Anan Y Wilson TD Gilbert DT. The feeling of uncertainty intensifies affective reactions. Emotion. (2009) 9:123. 10.1037/a0014607 19186925

[53] Hirsh JB Kang SK. Mechanisms of identity conflict: Uncertainty, anxiety, and the behavioral inhibition system. Personal Soc Psychol Rev. (2016) 20:223–44. 10.1177/1088868315589475 26048875

[54] Li J. The mechanism of why self-control resources and cognitive resources influence each other: an integrated model. Adv Psychol Sci. (2013) 21:235–42. 10.3724/SP.J.1042.2013.0023537113526

[55] Smith BM Twohy AJ Smith GS. Psychological inflexibility and intolerance of uncertainty moderate the relationship between social isolation and mental health outcomes during COVID-19. J Cont Behav Sci. (2020) 18:162–74. 10.1016/j.jcbs.2020.09.005 32953435 PMC7489247

[56] Chirkowska-Smolak T Garbacik Ż Piorunek M. Burnout syndrome in students and academic adjustment areas. An empirical context. Stud Theory Educ. (2022) 13:197–217. 10.5604/01.3001.0016.1133

[57] Pérez Fuentes MDC Molero Jurado MdM Simon Marquez MdM Oropesa Ruiz NF Gázquez Linares JJ. Validation of the maslach burnout inventory-student survey in Spanish adolescents. Psicothema (2020) 32:444–51. 10.7334/psicothema2019.373 32711681

[58] Ewert C Vater A Schröder-Abé M. Self-Compassion and coping: a meta-analysis. Mindfulness. (2021) 12:1063–77. 10.1007/s12671-020-01563-8

[59] Huang M Jing T Zhao S. The influence of college students' psychological inflexibility on depression: the role of ruminative responses and mindfulness. Psychol Res. (2019) 12:469–76.

[60] Fredrickson BL. The role of positive emotions in positive psychology: The broaden-and-build theory of positive emotions. Am Psychol. (2001) 56:218–26. 10.1037//0003-066X.56.3.218 11315248 PMC3122271

[61] Zhao Y Shi L Jiang Z Zeng N Mei H Lu Y. The phenotype and prediction of long-term physical, mental and cognitive COVID-19 sequelae 20 months after recovery, a community-based cohort study in China. Mol Psychiat. (2023) 28:1793–801. 10.1038/s41380-023-01951-1 36690792 PMC9869317

[62] Compuesto KM Bantog J Malabay GM Santibanez AM Tus J. Amidst the online learning modality: the self-efficacy and its relationship to the academic burnout of senior high school students. Psychol Educ. (2022) 1:174–84. 10.5281/zenodo.6654318

